# Axial resin duct quantification in tree rings: A functional defense trait

**DOI:** 10.1016/j.mex.2020.101035

**Published:** 2020-08-19

**Authors:** Sharon M. Hood, Charlotte C. Reed, Jeffrey M. Kane

**Affiliations:** a UDSA Forest Service, Rocky Mountain Research Station, Missoula, MT, United States; b Department of Forestry and Wildland Resources, Humboldt State University, Arcata, California, USA

**Keywords:** Duct, Canal, Tree defense, Wood anatomy, Tree resistance, Oleoresin, Xylem, Dendroecology

## Abstract

Resin ducts in the secondary xylem of tree rings are a measure of a tree's defense capacity from insects and pathogens. Because resin ducts are permanently embedded within the xylem, retrospective analysis can be performed to quantify changes in defense over time and determine factors that contribute to this change, such as climate and disturbance. Here, we provide methods on how to measure axial resin ducts in secondary xylem. These methods provide the necessary protocols for consistent quantification of xylem resin ducts and terminology, which will also allow easier cross-comparison among studies in the future. We describe:

• Steps to prepare tree cores for resin duct measurements.
• Procedure to obtain image and measure individual resin ducts.
• Software code to compile duct measurements into a complete chronology with both standardized and unstandardized resin duct metrics for further analyses.

## Graphical abstract

**Figure:**
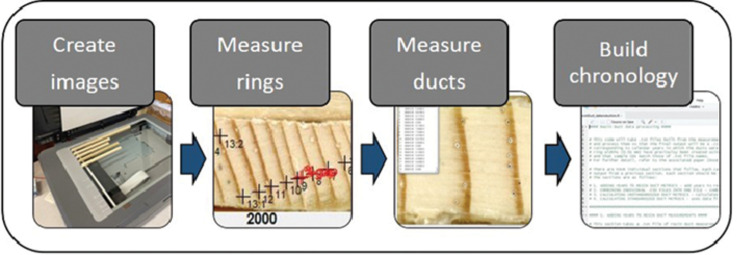

Specifications table

**Table:** 

Subject Area	Environmental Science
More specific subject area	Forest ecology, plant defense, dendroecology
Method name:	Axial resin duct quantification in tree rings
Name and reference of original method	Hood, S., and A. Sala. 2015. Ponderosa pine resin defenses and growth: metrics matter. Tree Physiology 35:1223-1235.
Resource availability	https://github.com/jeffkane/resinduct Hood, S.M., Reed, C.C., Kane, J.M. 2020, R code for axial resin duct quantification in tree rings, v1.0, Zenodo, doi: DOI: 10.5281/zenodo.3863014

## Background

Conifers have numerous constitutive and induced defenses to increase fitness from insects and pathogens [5,6]. A major defense is oleoresin or resin, which is a complex mixture of terpenoid compounds that acts as both a physical and chemical defense [11]. In *Pinus* species, resin is synthesized and stored in specialized structures called resin ducts or canals [1,17]. Axial and radial ducts form an interconnected network that deliver resin to wounding sites [3]. As sites of resin synthesis, storage, and delivery, resin ducts in the secondary xylem of tree rings are a measure of a tree's defense capacity from insects and pathogens.

**Fig. 1:**
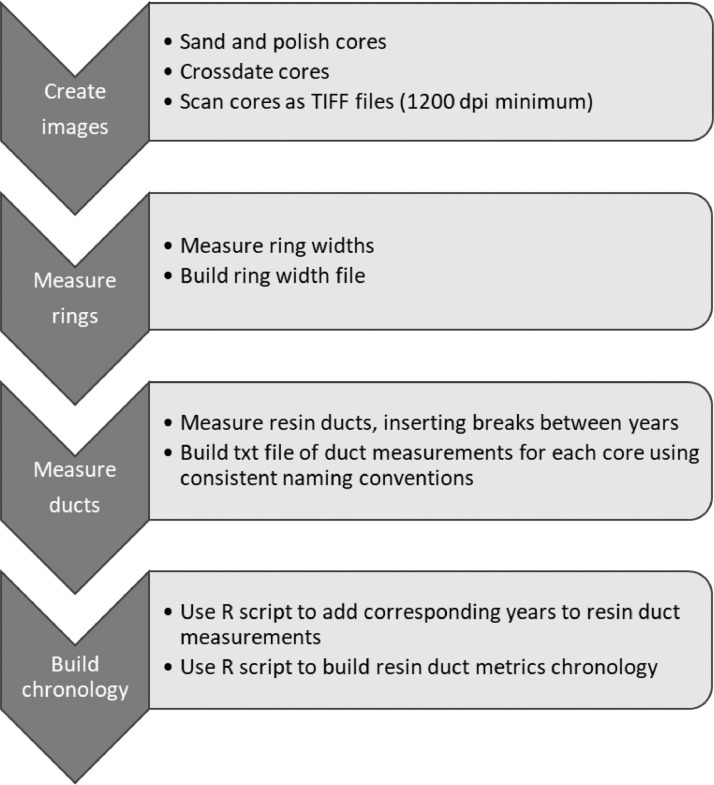
General workflow of measuring axial resin ducts in tree secondary xylem to build a master chronology of standardized and unstandardized metrics.

Because resin ducts are permanently embedded within the xylem, retrospective analysis can be performed to quantify defense and examine temporal changes due to climate and disturbance [7,^8^,^10^,[12], [13], [14], as well as interspecific comparisons [2,4]. Here, we provide methods on how to quantify axial resin ducts in secondary xylem. These methods provide the necessary protocols for consistent terminology and quantification of xylem resin ducts, which will also allow easier cross-comparison among studies in the future. We describe the basic work flow to build a resin duct chronology, including tree core preparation, procedures to image and measure individual ducts, and software code to compile duct measurements into a complete chronology with both standardized and unstandardized duct metrics for further analyses (Fig. 1).

## Core collection and preparation for measurement

Our methods focus on quantification of xylem resin ducts and assume the reader has knowledge of tree core collection and processing. We refer readers to Stokes and Smiley [16] and Speer [15] for details on collecting, mounting, preparing, and dating tree cores, but provide tips that are particularly relevant to measuring resin ducts here.

*Increment borer size*: When coring trees or sampling a tree section, the objective is to collect a large enough wood sample such that you have very few rings with no ducts to limit the number of zeros for the statistical analyses. We recommend collecting two cores per tree if using an increment borer that is 5.15 mm in diameter. One core is likely sufficient if using a 10 or 12 mm borer. The larger sized borer is also helpful if you are sampling trees that have been dead for a number of years and contain some rot.

*Core storage*: For 5.15 mm cores, use sturdy paper straws, such as Aardvark (Aardvark Straws, Ft. Wayne, IN, USA), to allow cores to air dry and prevent mold growth. These straws will not disintegrate in light rain and are easy to write on, yet still pliable enough to crimp the ends or join together to contain longer corers. After completing a plot or day, use newspaper to wrap the straws together to safely store them without danger of bending. Again, it is important to use permeable material to allow cores to dry and not mold. One benefit of bundling samples by plot or day is that mistakes in writing core data on the straws (e.g. tree ID, date, plot) can often be resolved based on the other cores in the bundle. For the larger 10–12 mm diameter cores, 12.6 mm (0.5 in) PVC pipe can be used to store cores with masking tape applied to the ends to secure and label the cores. Small drill holes can be added to facilitate drying and prevent mold. Drying cores for resin duct measurement is consistent with standard tree core preparation techniques. Air drying of cores typically takes approximately one week, but will vary by the humidity and temperature of the storing environment. Oven-drying is an option if time is limited, but it is not necessary. If oven-drying, the cores can be kept in their paper straws or PVC pipes in a 50–60 ^°^C oven for 1-2 days. Cores do not need to be completely devoid of moisture, just dry enough so that no shrinkage occurs after mounting.

*Mounting*: After cores are dried, they are ready to be mounted. It is important to orient the cores correctly such that the transverse plane or cross-section is facing up. Cores mounted incorrectly cannot be used to measure axial resin ducts. Look for the jagged lines on either side of the core – these are from tracheids being ripped in half lengthwise – and should be on the core sides when mounted. If the jagged lines referred to are not immediately visible, the core can be held in two hands and rolled back and forth. The ripped tracheids on either side of the core should reflect light and “glimmer”. One can also look at the end of the core to examine whether the tracheids are oriented vertically. As it is imperative that cores be mounted correctly, we direct readers unfamiliar with mounting techniques to Stokes and Smiley [16] or Speer [15] for further detail.

**Fig. 2:**
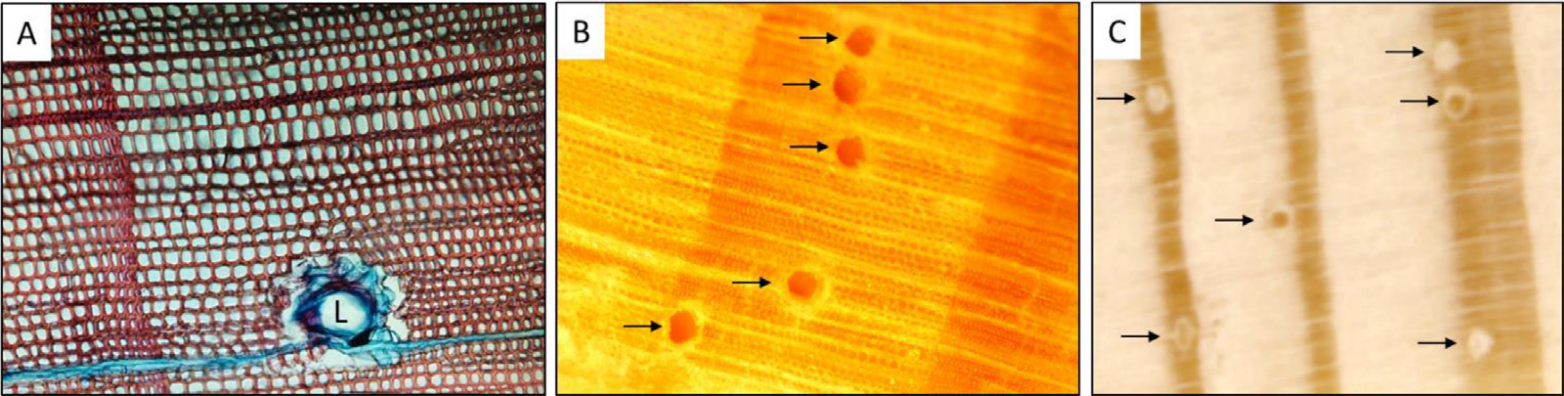
(A) Stained tissue cross section slide showing epithelial parenchyma cells surrounding an axial resin duct and ray parenchyma stained blue. L indicates the resin duct lumen. Tracheids are stained red. (B) Tree core prepared by sledge microtome. (C) Tree core prepared by sanding. Arrows in B and C show axial resin ducts embedded within annual rings.

*Surface preparation*: Surface preparation is particularly important for measuring resin ducts. Taking the time to prepare surfaces will save time later, as it is difficult to discern true resin ducts on surfaces that have been incorrectly prepared. In *Pinus* species, resin ducts are surrounded by thin-walled epithelial cells that synthesize resin and secrete it into the lumen for storage and delivery (Fig. 2A). Our method of axial resin duct measurement includes both the lumen and the epithelial parenchyma cells together and describes preparing tree cores by sanding. We recommend the sanding method for surface preparation when it is necessary to measure ducts over numerous years for many cores. Other methods such as tissue sections prepared for light microscopy allow more detailed measurement, such as number of epithelial cells and lumen area, but are infeasible for large sample sizes and are impractical when such detail is not needed. We have tried preparing cores by sledge microtome. The microtome has the advantage over sanding of not filling lumens with sawdust (Fig. 2B), but the resin creates a very shiny surface that makes it difficult to create a good image for measuring over a full tree core length. Sanding creates images that can be reliably measured over a whole core, but it does create variations in duct color, with some ducts getting packed with sawdust and some remaining open (Fig. 2C).

When sanding cores, the goal is to give each sample such a fine, flat surface that the cell structure is clearly visible when viewed under a dissecting scope. Rings should be clearly distinguishable on a well-prepared surface, with even very narrow rings visible to the naked eye. Sanding should be done using progressively finer grits. It is essential to not skip any of the grit levels. We recommend using a belt sander and a 120, 220, 320, and 400 grit progression. It is important to sand the cores evenly to create a flat surface and avoid a rounded edge for scanning. Afterwards, hand polish as needed with 40, 30, 15, and 9 micron polishing paper. The goal for each grit is to remove all the scratches from the previous grit. Use a hand lens or dissecting scope frequently while learning. When sanding samples from pines (*Pinus* spp.) in particular, resin may smear across the surface of cores if they become hot when sanding. It is best to minimize the possibility of this occurring by letting the cores and sander cool between grits and not sanding for prolonged periods of time. After sanding is finished, clean dust from the cores with compressed air.

*Crossdating*: Dating the core to have high confidence in the calendar year in which each ring formed is essential. Noting decadal years, missing or false rings with pencil marks during dating and prior to scanning will help keep your ring width and resin duct measurements lined up correctly. Again, consult Stokes and Smiley [16] or Speer [15] for more detailed information and strategies on crossdating.

## Image scanning

**Fig. 3:**
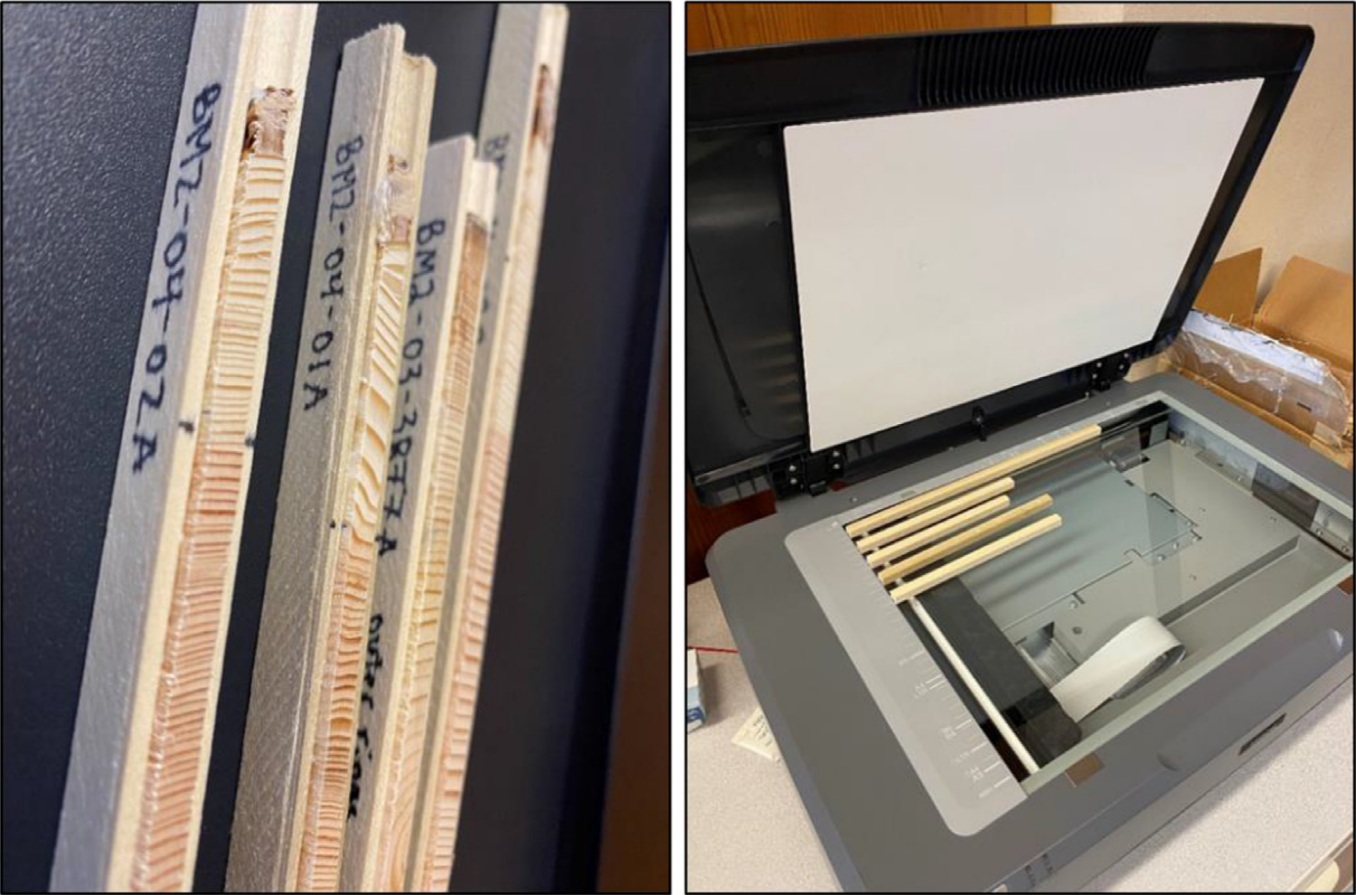
(A) Prepared, cleaned cores ready for scanning. (B) Cores placed on the scanner just before scanning.

Thoroughly clean the cores prior to scanning because any trace of debris will show up on the image greatly magnified (Fig. 3A). We suggest first using a piece of leather or chamois cloth to pull out remaining sanding dust on each core, followed by a blast of compressed air. Next make sure the scanner bed is free of dust; compressed air also works well here. Glass cleaner comes in handy to remove fingerprints.

**Fig. 4:**
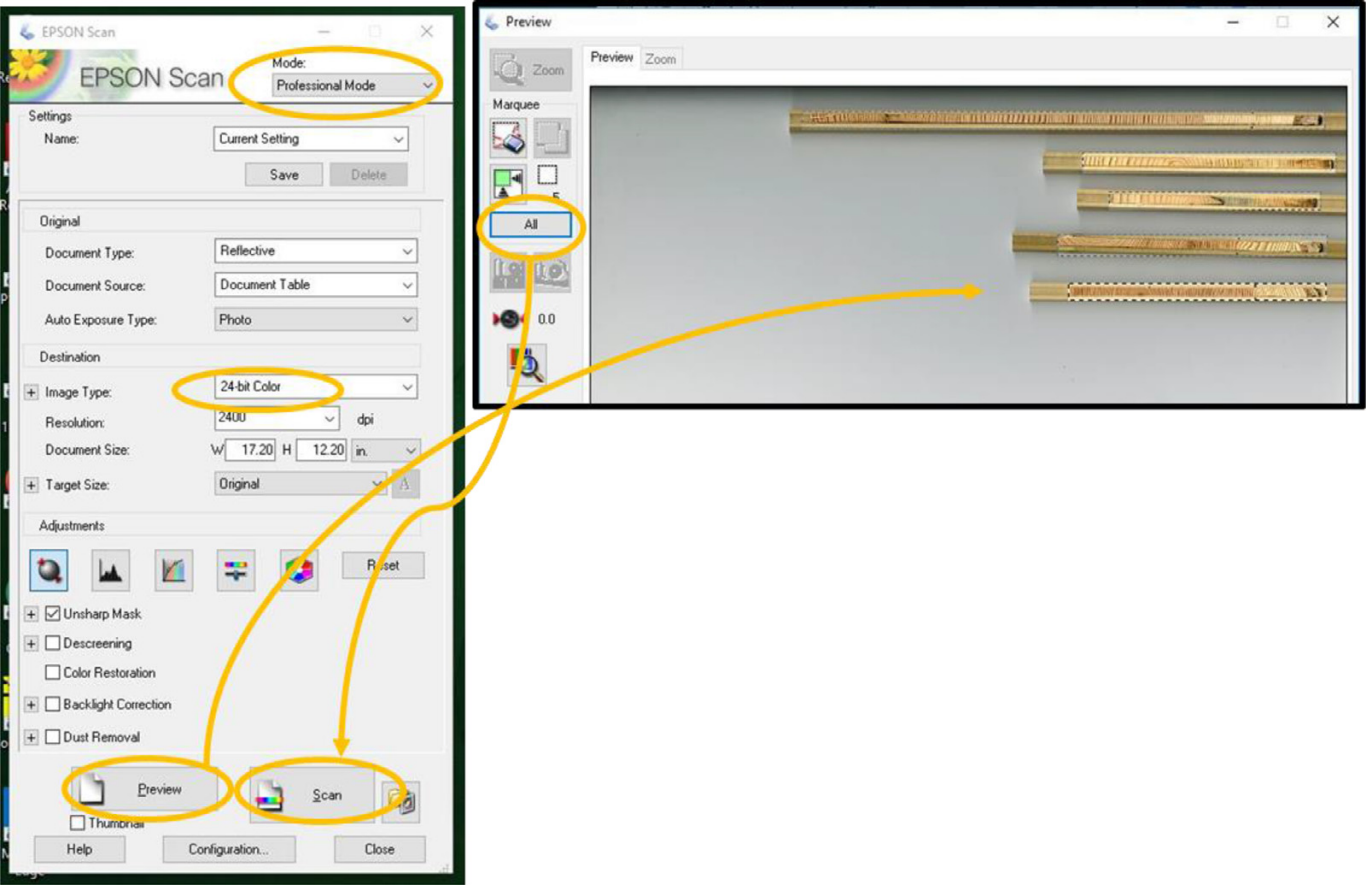
Screenshots of scanning options when creating the images of tree cores.

Use a flatbed scanner with an optical resolution capability of at least 2400 DPI (e.g. Epson Expression 11000XL) to create high-quality images of the cores. Place the cores on the glass and carefully lower the cover to not move the cores. Several cores can be placed on the scanner at one time (Fig. 3B). If necessary, a weight (e.g. a heavy book) may be placed on the scanner lid to help hold samples in place when scanning. It helps to develop a system when handling cores to make each step go smoothly – decide on an orientation such as pith to the left side – and treat all cores the same way. Now the cores are ready for scanning. We use the following settings and steps to create an individual TIFF file of each core, but this may change slightly depending on the scanner brand and software version (Fig. 4):

• Adjust settings: In “Professional” mode, ensure resolution is at least 1200 DPI but not more than 2400. Scanning at 1200 DPI is ideal as the time to scan and file size are far less than 2400 DPI, but if the core has particularly narrow rings, 2400 DPI may be necessary.
• Preview scan: Click the “Preview” button. A separate window will appear in which you can see a preview of your cores to image. Drag a box around the core(s) such that the entirety of the core(s) you are scanning is captured, while also making each box as small as possible. If scanning more than one core, click “All” after you have finished making a box around each core. These steps will crop your image(s) to the selected area to reduce scanning time and image file size(s). It will also create a separate image file for each core.
• Scan cores: In the main window, click the “Scan” button. A dialog box will pop up providing options of where to save your files and how to name them. Select the directory of your choice.
• Saving scanned files: Make sure files are saved as .tif and to follow naming conventions as described below. Scanning as TIFF files prevents image distortion and allows for the DPI to be automatically read into ImageJ.
• Click “OK”. A warning box may appear about the time needed to scan large images. Select “continue” and scanning will begin. Take a screen break, this will take several minutes.
• Adjusting images: After scanning is complete, images can be aligned and cropped with further precision if necessary in Adobe Photoshop or an equivalent photo-editing program. The final images should be named with a unique identifier and in a consistent, intuitive way. See note about file naming below.

Important note: Consistent file management and file naming is imperative from this step onwards or the R script described later will not work. Our R script requires using the naming convention that follows the Tucson format in which sample IDs (i.e., series ID) are limited to 8 characters and in which ring width measurements are stored in RWL files. For example: letter site ID, core number, core ID: SNF383La.tif, CA15a.tif. It is important for sample IDs in RWL files to be consistent with names of individual TIFF files created in this step.

## Ring width measurements

Ring width measurements are necessary for calculating resin duct metrics from tree cores. Any tree ring software will work to measure ring widths and create a ring width chronology. We use CooRecorder for measuring ring widths and CDendro for creating ring width chronologies (Cybis Elekronik & Data AB, Saltsjöbaden, Sweden). We additionally use COFECHA to check crossdating accuracy (Holmes 1983). The output from this step should be a RWL file. We explain steps directly relevant to measuring resin ducts here, but not the basics of using the software.

When opening TIFF files for ring measurement in CooRecorder, always double check that DPI is listed correctly (e.g. 1200 or 2400). Once ring widths for all cores within a chronology have been measured, they can be imported into CDendro where a ring width chronology (RWL file) can be built. CDendro gives the option to save ring measurements to 0.01 mm or 0.001 mm when creating RWL files in the Tucson format (see Settings > More settings); either will work. When saving a chronology as an RWL file in CDendro select either option to save as a Tucson .rwl file.

## Resin duct measurements

**Fig. 5:**
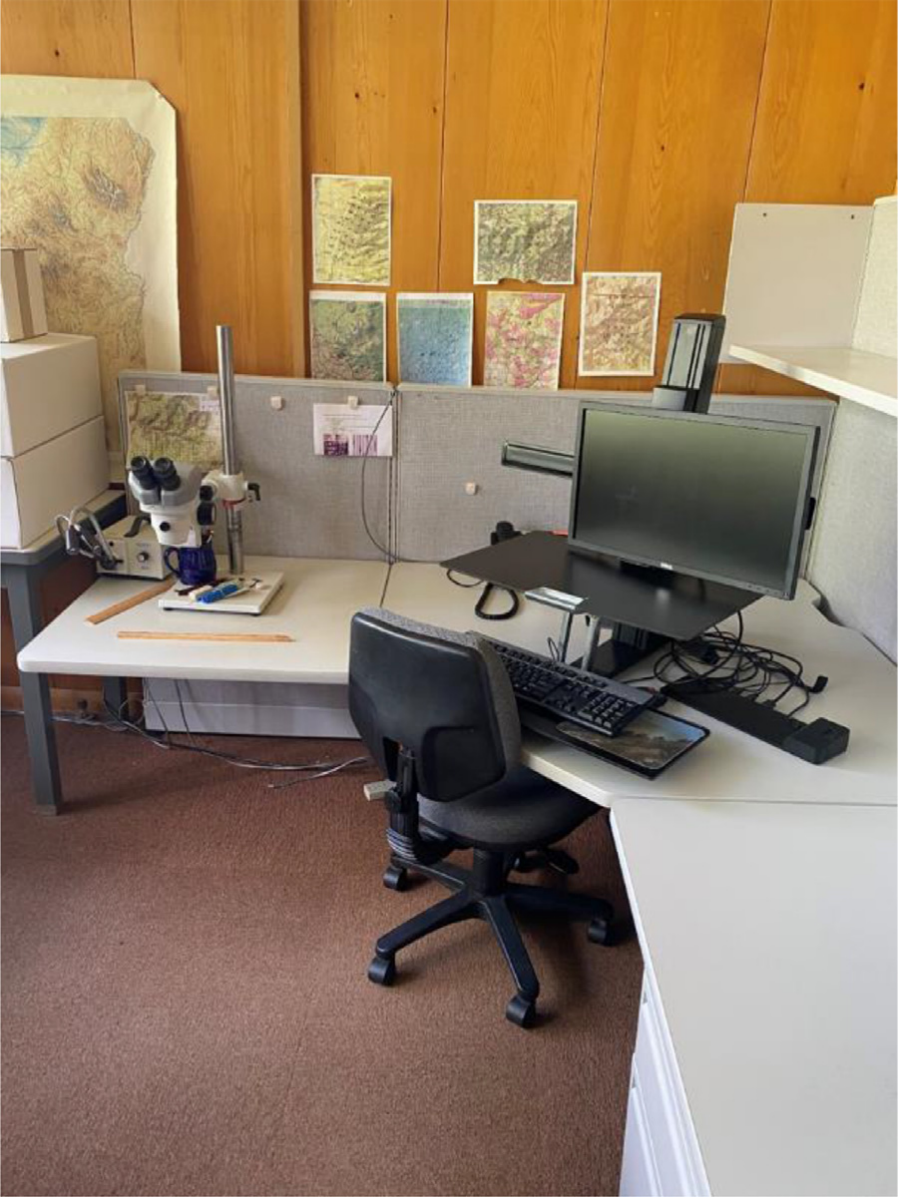
Example of a workstation prepared to measure resin ducts.

**Fig. 6:**
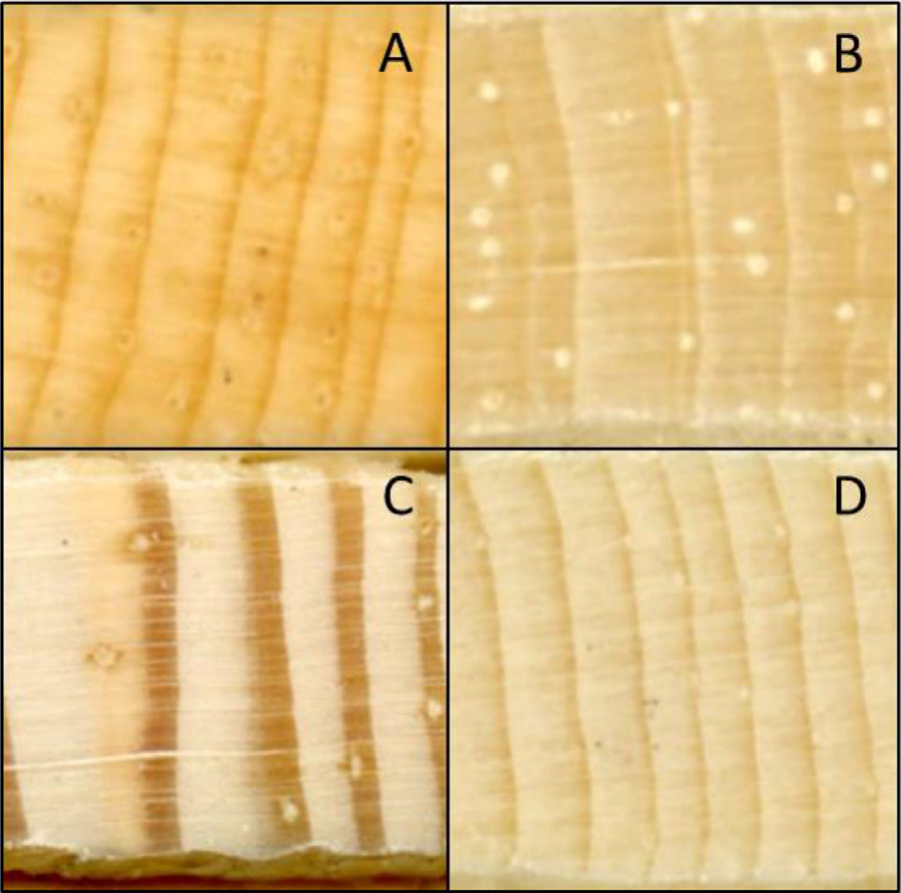
Examples of resin ducts from different pine species. (A) *Pinus monophylla*, (B) *P. aristata*, (C) *P. ponderosa*, (D) *P. contorta*.

For measuring ducts, you will need image processing software installed on your computer, a dissecting scope, and the cores all within easy reach (Fig. 5). The human eye can discern more detail and nuances using a dissecting scope than from a scanned image. We are unaware of a method to accurately detect and measure resin ducts automatically within an image. While such a program may be developed, it would be difficult to account for the high color variability within and among cores across multiple species and the user would still need to verify resin ducts visually by examining the tree core under a dissecting scope. Therefore, to accurately identify ducts, it is important to alternate between the image on the monitor and looking at the core under the scope. Before you start measuring ducts, we recommend spending time to look at many of the cores to see the variation and get a feel for what resin ducts look like. Appearances can change by species (Fig. 6).

We recommend the image processing software ImageJ (National Institutes of Health, Bethesda, MD, USA); the methods described likely will need to be modified if other software is used. ImageJ is free software available via the National Institutes of Health (https://imagej.nih.gov/ij/) that allows measurement of individual resin duct areas within tree rings, as well as creation of an image with an overlay of the duct measurements for quality control and a text file of the resin duct areas. When using ImageJ for the first time, set options to the following:

• Analyze > Set Measurements > check boxes: Area, Add to Overlay, Display label
• Verify that scale is correct using Analyze>Set Scale: check that the correct DPI is displayed
• Choose the preferred colors for the duct outline and label by going to Image>Overlay>Labels…

**Fig. 7:**
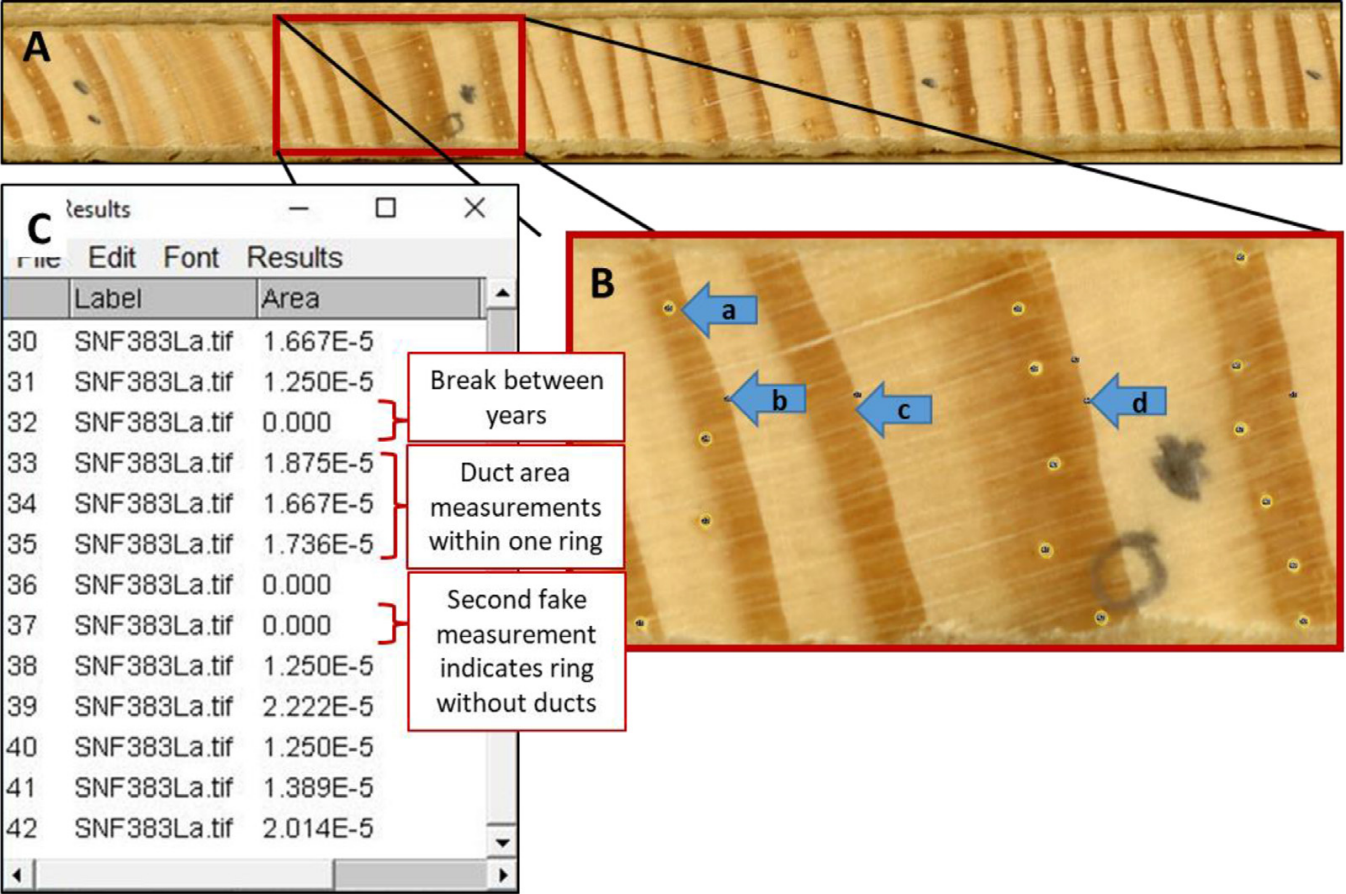
(A) Sanded, crossdated and scanned core. (B) Section of core with resin ducts outlined using ImageJ. (a) Resin duct outlined and measured, (b) Fake (i.e. ‘zero’) duct measurement between rings, (c) Ring has no resin ducts therefore only a zero is included, (d) A second zero has been added because of a missing ring. (C) Example of ImageJ measurement window for the same core with duct area measurements.

You are now ready to start measuring ducts following the steps below. See Fig. 7 for an example of a core with the ducts outlined using ImageJ to get an idea of what the process will look like.

• Open image of core by going to File > Open and navigating to the directory with the core images
• Immediately save this file as a new file TIFF in a subdirectory named “labelled cores” and add a “_label” to end of file name. This will save a file with ducts outlined and numbered as a TIFF and can be used for quality control purposes (DO NOT OVERWRITE ORIGINAL IMAGE)
• Zooming to 150-200% typically works best to see ducts
• Start with oldest year (towards pith) and move to recent years (toward bark)
• Measure ducts with the Ellipse tool
• Draw ellipse that best fits the duct; size and shape of ellipses can be adjusted once drawn. As noted above, resin ducts can vary in appearance throughout the core. Each duct measurement includes both the lumen and the epithelial parenchyma cells surrounding the lumen.
• After you are satisfied with the outline, press ‘m’ to measure and record area (inches^2^) of each duct. You will see a box with “Results” on the top pop up – this will start a file of the measurements and add a border and id to the measured duct on the image (Fig. 7C).
• After measuring all ducts in one year (ring), insert a “fake” measurement by clicking image near ring boundary and measuring. This will create a line in the TXT file with a zero value for area so that the R code can assign correct years. Always double check that the fake measurement has indeed been recorded as a zero in your measurement window (Fig. 7C).
• If a ring has no resin ducts, insert a fake measurement at the ring boundary before moving on to the following ring (Fig. 7B-c)
• Always be aware of where missing rings occur on the core being measured. It is helpful to mark these on the core itself or have the corresponding ring width file open in a separate window in order to ensure missing rings do not get missed. When a missing ring occurs, add a second fake measurement along the ring boundary where the missing ring falls (Fig. 7B-d).
• If a resin duct falls along a crack, make it a little smaller to account for the crack stretching out the image
• Check the physical core under a microscope for every ring to verify the image as ducts can be difficult to discern even on well-prepared surfaces
• Ducts can be right next to each other – measure them individually
• After all ducts are measured for the core, save the file of duct measurements by clicking File>Save As in the Results box. Navigate to the desired directory and name the file as sampleID.txt. ImageJ will default to saving measurements as CSV files so it is important to include the .txt file extension when saving measurements. Close the Results box. *Again, the sample ID used in the TXT file name must match the sample ID of the RWL file in order for the R script to run effectively*.

## Adding calendar years to duct measurement

After all resin ducts in the core are measured, the next step requires adding the calendar year of formation to each duct. We recommend doing this step immediately after measuring ducts for each core so that the individual core is fresh in your mind. For example, you'll know if there were any rings with zero ducts or missing rings that could cause misalignment of years, or maybe you measured something and then decided it was not a duct after all. These errors are relatively simple to fix in the TXT file.

The latest version of the R script described below and referenced in these steps can be found at https://github.com/jeffkane/resinduct [9].

The R script will add years to each row using the following steps:

• Open R script; Section 1 contains code for adding years
• Required user-defined variables file location, output file name, and the year of the inner-most (oldest) ring in which resin ducts have been measured.
• Run Section 1
• The output will be a CSV file with years added and will be saved to the specified file location
• Open to verify years are assigned correctly, paying extra attention to years with zero ducts or where missing rings occur

## Building final chronology of resin duct metrics

Once years have been added to resin duct measurements for all cores of interest, your directory should include individual CSV files corresponding to individual cores with duct measurements and associated calendar years.

• Section 2 of the R script will import these individual CSV files and compile them into a new CSV file containing years and duct area measurements for all cores.
  ○ Required user-defined variables: file location and output file name
• Section 3 of the R script computes unstandardized resin duct metrics (Table 1) using the CSV file created in Step 2.
  ○ Required user-defined variables: file location and output file name

**Table 1:** Resin duct metric name and description.

Metric	Type^1^	Description
Duct size (mm^2^)	Unstandardized	Mean size of all ducts per annual ring
Duct production (no. year^−1^)	Unstandardized	Total number of ducts per annual ring
Total duct area (mm^2^ year^−1^)	Unstandardized	Sum of duct area per annual ring
Duct density (no. mm^−2^ year^−1^)	Standardized	Total number of ducts per annual ring divided by ring area^2^
Relative duct area (% annual ring)	Standardized	Total duct area divided by ring area^2^ × 100

1 Standardized = adjusted for growth rate; unstandardized = unadjusted for growth rate.

2 Ring area = ring width × core diameter.
• Section 4 of the R script computes standardized resin duct metrics (Table 1) and requires 1) the CSV file of unstandardized metrics created in Step 3, and 2) the ring width chronologies (or individual files) for the same trees for which resin duct measurements were performed (i.e. RWL files). RWL files should be placed in the same directory as the CSV file of unstandardized metrics prior to executing this section. The code computes ring area using the RWL files and the average width of the core (e.g. 5.15 mm). Core width is easy to obtain using the straight line measuring tool in ImageJ or by using the inside diameter of the increment borer. If users want to use ring-level core width measurements to calculate ring area, they will need to modify the provided code. This may be necessary if a core is partially damaged.
  ○ Required user-defined variables: file location, the first and last years for which resin ducts were measured, and the average core width.
• The final output is a CSV file with five resin duct metrics as well as ring width (mm) and ring area (mm^2^) associated with calendar years for each core which can be used for desired statistical analyses.
• Section 4.1 of the R script is optional and allows for multiple cores from the same tree to be added together (e.g. A and B cores). In particular, when using 5.15 mm cores, taking two cores reduces the number of zeros in the final resin duct metric chronology. This section of script assumes that core ID (e.g. A or B) falls at the END of the sample ID character string (e.g. SNF383La).
  ○ Required user-defined variables: output file name

## Method validation and quality control

We find it easiest to conduct quality assessments at each major step rather than waiting until the end. An accurate ring width file is the foundation for building the final resin duct chronology and time should be spent to ensure that all cores included are datable for the time period of interest. As noted above, we recommend adding years to each resin duct file immediately after duct measurement is completed for an individual core rather than in batches, and have thus set up Section 1 of the R script to function in this way. This will reduce time spent correcting errors in years not aligning correctly because the image of the core in question is still fresh in your mind. If an error in years occurs, you can either correct it by hand in the TXT file or redo the resin duct measurements for that core. We find it easier to correct the TXT file by inserting year breaks, deleting double counted ducts, etc. rather than starting over in ImageJ. After the master resin duct chronology is created, basic data plotting will allow checking for implausibly large or small values.

We encourage archiving data in a publicly accessible repository such as the International Tree-Ring Databank (ITRDB; https://www.ncdc.noaa.gov/data-access/paleoclimatology-data/datasets/tree-ring). The ITRDB now allows inclusion of resin duct chronologies.

## Supplementary material and/or additional information

The latest version of the R script described in the paper can be found at https://github.com/jeffkane/resinduct [9].

## Declaration of Competing Interest

The authors declare that they have no known competing financial interests or personal relationships that could have appeared to influence the work reported in this paper.
